# Neurodiversity Within an Adult Home Treatment Team in Newham, London

**DOI:** 10.1192/bjo.2022.461

**Published:** 2022-06-20

**Authors:** Johnny Iyiola, Fergus Lewis, Stephanie Glavin, Richa Shukla

**Affiliations:** East London NHS Foundation Trust, London, United Kingdom

## Abstract

**Aims:**

An increasing proportion of patients presenting in crisis to Newham Home Treatment Team (NHTT) had been noted to exhibit clinical signs and symptoms of neurodiversity. The aim of our project was to identify the number of confirmed and suspected autism and ADHD cases over a 12 month period. We also collated data on gender, age, presenting complaint, medication, and use of screening tools.

**Methods:**

The project involved a retrospective case note review of the NHTT (South) caseload from November 1st 2020-October 31st 2021. This involved searching caseload and electronic patient records on RiO for keywords: “autism”, “ADHD”, “ASD”, “Asperger”, “Attention Deficit Hyperactivity Disorder”, “AQ10”. Patients were included if neurodiversity was suspected or already diagnosed. Data were collected on age at presentation, gender, presenting complaint, NHTT diagnosis, other diagnoses, Autism Spectrum Quotient (AQ-10) score, whether screening for attention deficit hyperactivity disorder (ADHD) was completed, age at first presentation to services, and medications at discharge.

**Results:**

Over a 12 month period 49 patients (out of 258) presenting to NHTT South raised clinical suspicion of neurodiversity, representing 19% of the caseload and on average one new patient per week. The majority of these (47) related to autism, 13 of which had confirmed diagnosis (M = 26, F = 23). Of the 36 for whom there was clinical suspicion of autism, an AQ10 score was recorded for 18. 14 patients were suspected to have ADHD, 6 of which were confirmed (M = 5, F = 9). There was not a significant impact of gender. The majority of patients included (33) presented with a mood disorder (M = 15, F = 18), and a minority (13) with psychotic disorders (M = 7, F = 6). Over half of patients included presented with suicidality (M = 11, F = 14), and just under half had received a diagnosis of personality disorder (M = 7, F = 16). 21 patients were prescribed anti-psychotic medication (M = 13, F = 8), and 24 were prescribed an antidepressant (M = 9, F = 15).

**Conclusion:**

Our findings demonstrate that neurodiversity may currently be under-diagnosed and is often mis-diagnosed, with suspicion frequently raised in previously undiagnosed adult psychiatric patients presenting in crisis. There is need for increased awareness of presenting features of neurodiversity within secondary mental health care services, and particular screening for patients experiencing suicidality may be beneficial. The AQ10 is under-utilised as an independent screening tool, and should be promoted to aid in identifying neurodiversity. Patients who may be neurodiverse are frequently prescribed antipsychotic and antidepressant medication, and further studies exploring prescribing practices in this patient sub-set may be useful.

